# Activation of amygdala prokineticin receptor 2 neurons drives the anorexigenic activity of the neuropeptide PK2

**DOI:** 10.1016/j.jbc.2022.102814

**Published:** 2022-12-17

**Authors:** Terry C. Yin, Ayushi Mittal, Paul Buscaglia, Wenxian Li, Julien A. Sebag

**Affiliations:** 1 Department of Molecular Physiology and Biophysics, University of Iowa, Iowa City, Iowa, USA; 2 Fraternal Order of Eagle Diabetes Research Center, University of Iowa, Iowa City, Iowa, USA; 3 Iowa Neuroscience Institute, University of Iowa, Iowa City, Iowa, USA; 4 Pappajohn Biomedical Institute, University of Iowa, Iowa City, Iowa, USA

**Keywords:** prokineticin, amygdala, MRAP2, PKR2, food intake, AAV1, adeno-associated virus 1, ARC, arcuate nucleus of the hypothalamus, A/P, anterior/posterior, CNO, clozapine *N*-oxide, D/V, dorsal/ventral, GPCR, G protein–coupled receptor, gRNA, guide RNA, ICV, intracerebroventricular, LA, lateral amygdala, M/L, medial/lateral, MRAP2, melanocortin receptor accessory protein 2, PK, prokineticin, PKR, prokineticin receptor, qPCR, quantitative PCR

## Abstract

Energy homeostasis is a complex system involving multiple hormones, neuropeptides, and receptors. Prokineticins (PK1 and PK2) are agonists to two G protein–coupled receptors, prokineticin receptor 1 and 2 (PKR1 and PKR2), which decrease food intake when injected in rodents. The relative contribution of PKR1 and PKR2 to the anorexigenic effect of PK2 and their site of action in the brain have not yet been elucidated. While PKR1 and PKR2 are both expressed in the hypothalamus, a central region involved in the control of energy homeostasis, PKR2 is also present in the amygdala, which has recently been shown to regulate food intake in response to several anorexigenic signals. PKR trafficking and signaling are inhibited by the melanocortin receptor accessory protein 2 (MRAP2), thus suggesting that MRAP2 has the potential to alter the anorexigenic activity of PK2 *in vivo*. In this study, we investigated the importance of PKR1 and PKR2 for PK2-mediated inhibition of food intake, the brain region involved in this function, and the effect of MRAP2 on PK2 action *in vivo*. Using targeted silencing of PKR2 and chemogenetic manipulation of PKR2 neurons, we show that the anorexigenic effect of PK2 is mediated by PKR2 in the amygdala and that altering MRAP2 expression in PKR2 neurons modulates the activity of PK2. Collectively, our results provide evidence that inhibition of food intake by PKs is not mediated through activation of hypothalamic neurons but rather amygdala PKR2 neurons and further establishes the importance of MRAP2 in the regulation of energy homeostasis.

Prokineticin, PK1 and PK2, are small proteins of about 9 kDa containing 10 cysteines that form five disulfide bonds. Whereas PK2 is expressed throughout the brain including the cortex, thalamus, hypothalamus, and amygdala, PK1 is largely restricted to the brainstem (1). These proteins act as agonists for two G protein–coupled receptors (GPCRs), prokineticin receptors 1 and 2 (PKR1 and PKR2). Whereas PKR1 is widely expressed in peripheral tissues, PKR2 is mostly expressed in the central nervous system. PKs were first identified as factors that promote intestinal smooth muscle contraction (2, 3). They were then shown to act on peripheral sensory neurons and enhance nociceptive sensitivity (4, 5, 6, 7, 8). More recently, central administration of PK2 was shown to significantly decrease food intake (9), thus identifying PKRs as central regulators of energy homeostasis. Whereas the role and mechanism of action of several GPCRs involved in the regulation of food intake expressed in the hypothalamus have been extensively studied, the role, regulation, and site of action of PKRs in the central nervous system are poorly understood. PKR1 and PKR2 have different distribution in the brain, and while both PKR1 and PKR2 are expressed in the arcuate nucleus of the hypothalamus (ARC), only PKR2 is expressed in the amygdala (1, 10), a brain region with an emerging important role in the control of energy homeostasis (11, 12). The relative contribution of PKR1 and PKR2 in the control of food intake is unclear, and the brain region responsible for the anorexigenic action of PK2 has not yet been identified.

PKR couple through both Gα_s_ and Gα_q/11_ proteins resulting in both cAMP and calcium downstream signaling (13). Both PKR1 and PKR2 are regulated by the melanocortin receptor accessory protein 2 (MRAP2) (13), a single transmembrane protein that was previously demonstrated to alter the trafficking and signaling of multiple GPCRs involved in the regulation of energy homeostasis (14, 15, 16, 17, 18, 19, 20, 21). MRAP2 is essential for ghrelin receptor function (19, 20), potentiates signaling of the melanocortin-4 receptor (14, 18), but inhibits orexin receptor 1 and PKRs (22). In the presence of MRAP2, PKR1 and PKR2 are retained in the endoplasmic reticulum and consequently cannot be activated by PK2 (13, 22). The retention of PKRs in intracellular compartments appears to be due to MRAP2 preventing N-linked glycosylation of receptors (23, 24). *In vivo*, global deletion of MRAP2 results in the enhancement of the anorexigenic effect of PK2 (13), consistent with MRAP2 being an endogenous inhibitor of PKRs. In this study, we identify PKR2 as the receptor responsible for the PK2-mediated inhibition of food intake, demonstrate that the anorexigenic effect of PK2 is mediated by PKR2 neurons located in the lateral amygdala (LA) rather than hypothalamic neurons, and that the modulation of MRAP2 expression in LA^PKR2^ neurons alters the effect of PK2 on feeding. Results from this study identify PKR2 as a possible new target for the development of drugs for obesity.

## Results

### The orexigenic action of PK2 is mediated through PKR2

**Figure 1:**
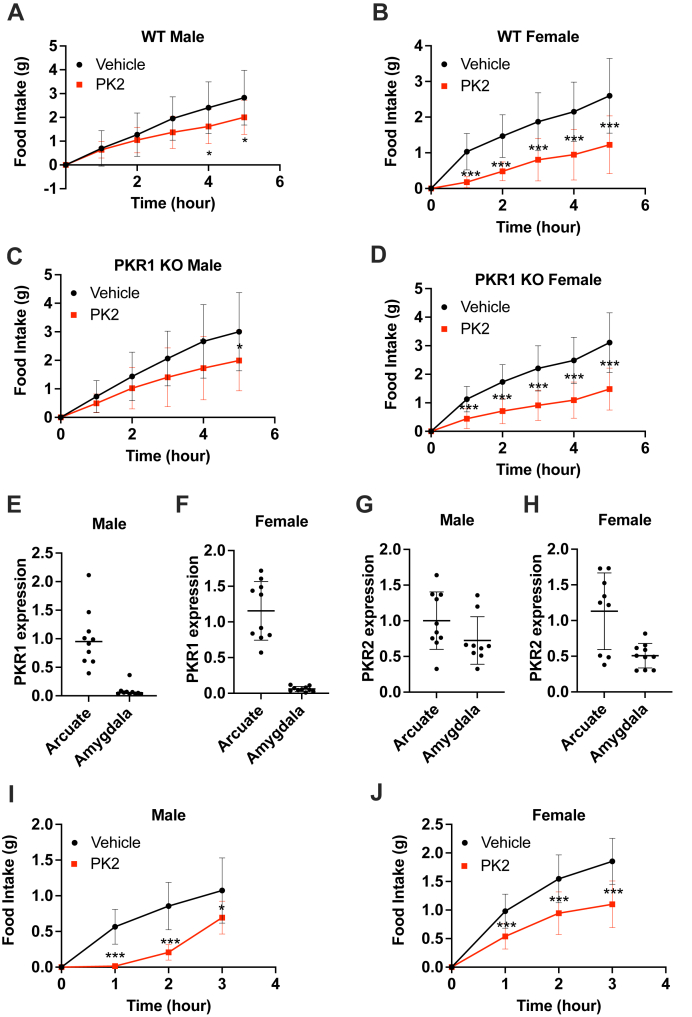
**PKR1 is not required for PK2-mediated feeding behavior.***A*–*C*, food intake after overnight fast and central administration of vehicle (*black*) or 0.65 μg PK2 (*red*) in male (n = 18) and female (n = 25) WT (*A* and *B*) or male (n = 18) and female (n = 27) *Pkr1* KO mice (*C* and *D*). *E* and *F*, qPCR measurement of PKR1 expression in the arcuate nucleus and amygdala of male (*E*) and female (*F*) mice (n = 9–10 per group). *G* and *H*, qPCR measurement of PKR2 expression in the arcuate nucleus and amygdala of male (*G*) and female (*H*) mice (n = 9–10 per group). *I* and *J*, food intake in overnight fasted male (n = 11) and female (n = 15) mice after injection of vehicle (*black*) or 0.65 μg PK2 (*red*) unilaterally into the amygdala. Results are mean ± SD. ∗*p* < 0.05, ∗∗*p* < 0.01, and ∗∗∗*p* < 0.001. PK, prokineticin; PKR, prokineticin receptor; qPCR, quantitative PCR.

Central injection of PK2 decreases food intake; however, the receptor involved in this action of PK2 has not been clearly identified. Previous studies hypothesized that the anorexigenic action of PK2 was mediated through the activation of PKR1, which is expressed in pro-opiomelanocortin neurons of the ARC (9). To test the requirement of PKR1 for PK2 to inhibit food intake, we generated a *Pkr1* KO mouse model using CRISPR–Cas9 technology. This model was engineered as a “KO first,” meaning it can be converted to a conditional Pkr1 (floxed) mouse through breeding with a mouse expressing flippase. This was achieved by inserting a cassette containing an FRT-flanked Nluc followed by a LoxP-flanked inverted mCherry between the second and third exons of the *Pkr1* gene (Fig. S1*A*). Proper insertion of the cassette was verified by sequencing, and loss of PKR1 expression was verified by quantitative PCR (qPCR) (Fig. S1*B*). Male and female WT and *Pkr1* KO mice, cannulated in the lateral ventricle and habituated to home cages of a BioDAQ automated food intake recording instrument, were fasted overnight and injected with saline or 0.65 μg PK2 intracerebroventricular (ICV) before providing them with food. As previously shown, PK2 injection significantly reduced food intake compared with vehicle in both male and female WT mice (Fig. 1, *A* and *B*). Interestingly, the orexigenic effect of PK2 was fully retained in *Pkr1* KO mice (Fig. 1, *C* and *D*), thus demonstrating that PKR1 is not required for the anorexigenic activity of PK2.

Because of this result, we then investigated the role of PKR2 in regulating food intake. Like PKR1, PKR2 is expressed in the ARC; however, in contrast to PKR1, PKR2 is also present in the amygdala (1, 10), a brain region with emerging roles in energy homeostasis. To verify the distribution of both receptors, we extracted RNA from micropunches of the ARC and from the amygdala and measured PKR1 and PKR2 expression by qPCR. In agreement with previous reports, we found that PKR1 mRNA was present in the hypothalamus but not detected in the amygdala (Fig. 1, *E* and *F*). In contrast, PKR2 mRNA was detectable in both brain regions (Fig. 1, *G* and *H*), confirming that only PKR2 is present in the amygdala. To test whether activation of PKR2 in the amygdala is sufficient to inhibit food intake, male and female mice were stereotaxically unilaterally implanted with a canula in the amygdala. After recovery, animals were fasted overnight and injected with saline or PK2 in the amygdala before providing them with food. Local injection of PK2 in the amygdala significantly decreased food intake compared with control in both male and female mice (Fig. 1, *I* and *J*), thus suggesting that stimulation of PKR2 neurons in the amygdala is sufficient to mediate the anorexigenic action of PK2.

### PK2 regulates food intake through activation of PKR2 in the amygdala

**Figure 2:**
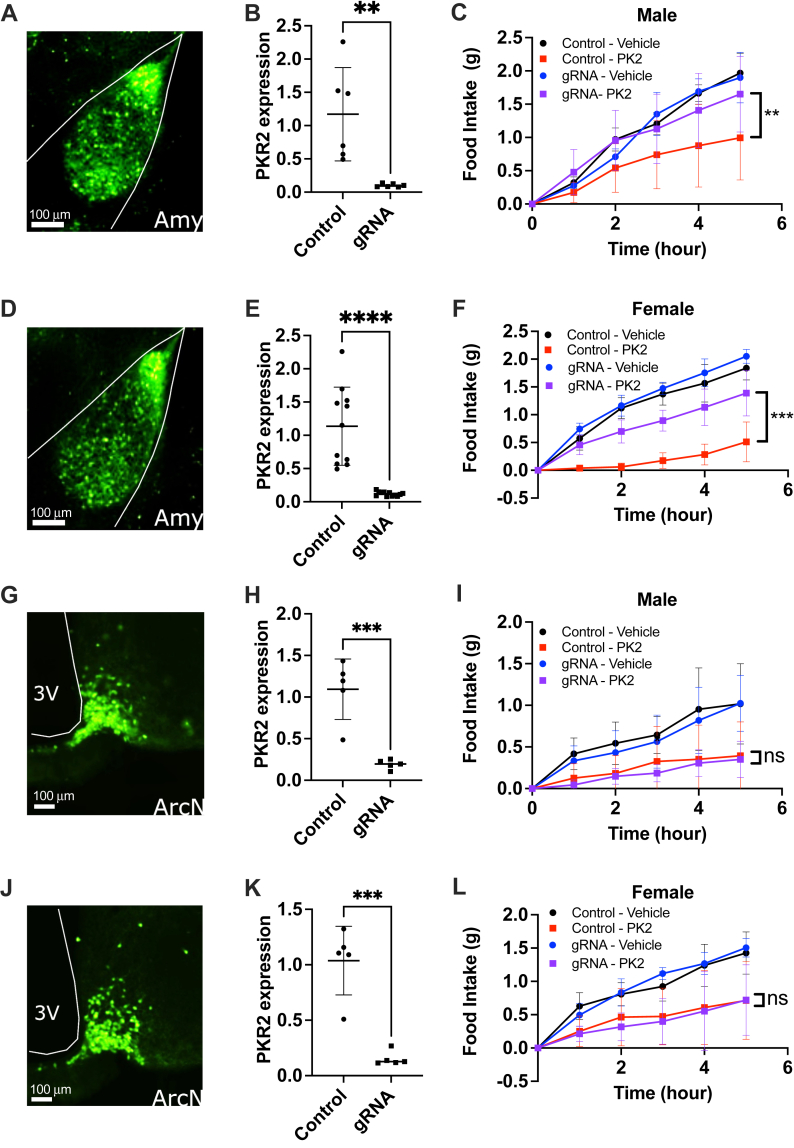
**PKR2 expression in amygdala neurons is required for PK2 activity.***A*, *D*, *G*, and *J*, amygdala (*A* and *D*) or arcuate nucleus (ARC) (*G* and *J*) targeting of control AAV-GFP virus in brain slices. *B*, *E*, *H*, and *K*, qPCR measurement of PKR2 expression in the amygdala (*B* and *E*) and the ARC (*H* and *K*) of mice injected with either control of PKR2 gRNA virus in the corresponding brain region (n = 5–6 per group). *C*, *F*, *I*, and *L*, food intake in mice bilaterally injected with either control of PKR2 gRNA virus in the amygdala or ARC following an overnight fast and ICV injection of vehicle or 0.65 μg PK2. Results are mean ± SD. ∗*p* < 0.05, ∗∗*p* < 0.01, and ∗∗∗*p* < 0.001. AAV, adeno-associated virus; Grna, guide RNA; ICV, intracerebroventricular; PK, prokineticin; PKR, prokineticin receptor; qPCR, quantitative PCR.

Global deletion of PKR2 results in serious developmental defects mimicking Kallmann syndrome (25). Consequently, *Pkr2* KO mice are ill fitted to assess the role of PKR2 in the regulation of feeding. For this reason, we opted to use the CRISPR interference technology to specifically silence PKR2 expression in the amygdala of adult animals. dCas9-KRAB mice ubiquitously express the catalytically deactivated Cas9 fused to the KRAB transcriptional repressor domain, thus allowing targeted and specific inhibition of gene transcription by injection of a virus coding for a guide RNA (gRNA). Male and female dCas9-KRAB mice received bilateral adeno-associated virus 1 (AAV1) injection in the LA or the ARC using either a control virus expressing GFP or a virus coding for two gRNAs targeting the first exon of PKR2. Animals were then cannulated in the lateral ventricle to allow for ICV injections. Proper targeting of the viral injections was verified by detection of GFP in coronal brain sections (Fig. 2, *A*, *D*, *G*, and *J*), and PKR2 silencing was measured by qPCR in micropunch samples from targeted regions. Injection of gRNA coding AAV1 resulted in an 85 to 90% knockdown of PKR2 expression compared with control (Fig. 2, *B*, *E*, *H*, and *K*). After the recovery period, mice were fasted overnight and injected centrally with saline or PK2 before measuring food intake. As expected, central PK2 injection in male and female mice infected with control virus in the LA resulted in a significantly decreased food intake compared with saline-injected controls (Fig. 2, *C* and *F*). In contrast, the anorexigenic effect of PK2 was almost completely abolished in animals following PKR2 silencing in the LA (Fig. 2, *C* and *F*). This result establishes the importance of LA PKR2 for the regulation of feeding by PK2. Importantly, silencing of PKR2 expression in the ARC did not impair the ability of central PK2 to inhibit food intake (Fig. 2, *I* and *L*), thus demonstrating that PKR2 expression in the ARC is not required for the anorexigenic action of PK2. The results of these experiments reveal that LA PKR2 rather than ARC PKR2 is essential for PK2-mediated inhibition of feeding.

### Acute inhibition of PKR2 increases feeding

**Figure 3:**
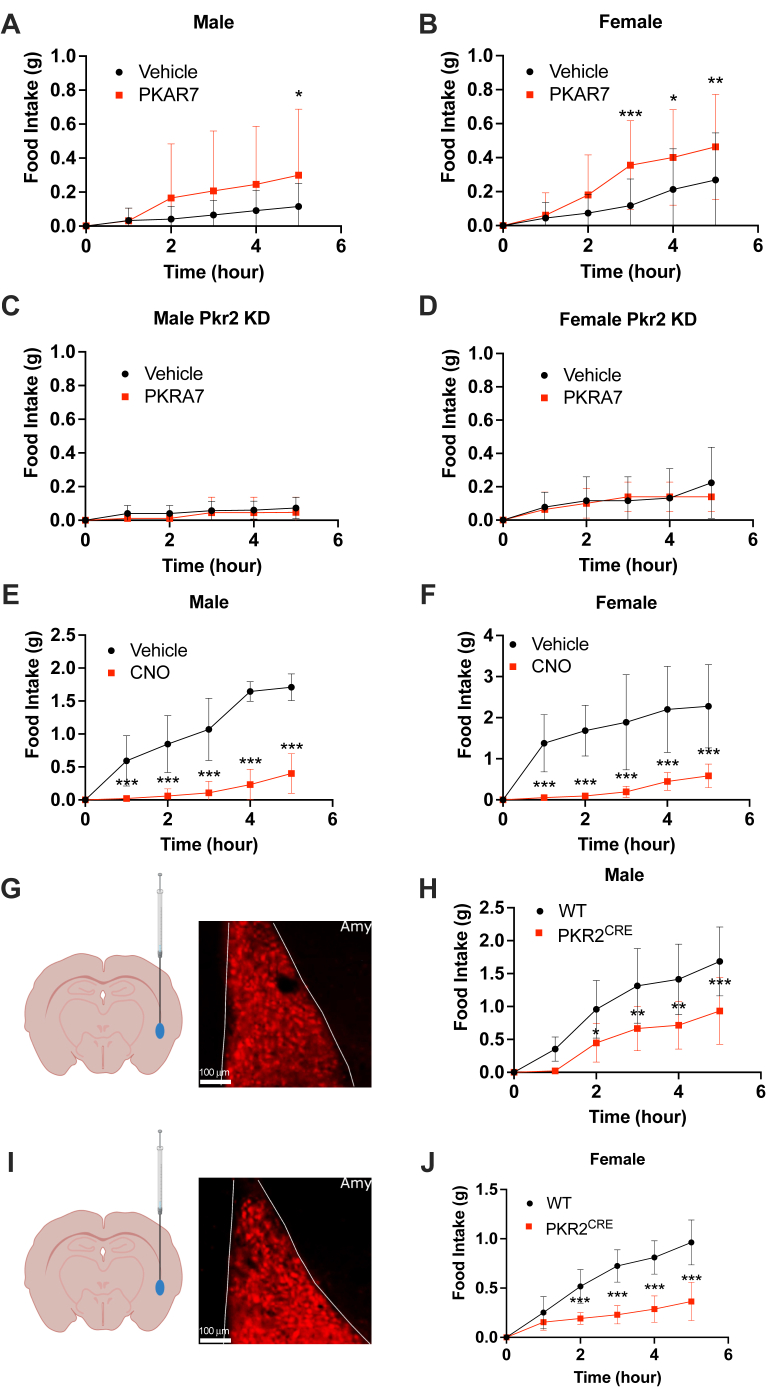
**Inhibition of PKR2 is orexigenic, and activation of PKR2 neuron inhibits feeding.***A* and *B*, food intake in sated male (*A*) and female (*B*) mice following ICV injection of vehicle or PKRA7 (n = 19–22 per group). *C* and *D*, food intake in sated male (*C*) and female (*D*) dCas9-KRAB mice injected bilaterally with AAV1-PKR2 gRNA in the amygdala following ICV injection of vehicle or PKRA7 (n = 5–7 per group). *E* and *F*, food intake after overnight fast and peripheral administration of vehicle or CNO in PKR2^CRE^ male (*E*) and female (*F*) mice transduced with AAV-PHP.eB-hSyn-DIO-hM3D(Gq) (n = 12 per group). *G*–*J*, targeting verification of AAV1-hSyn-DIO-hM3(Gq)-mCherry virus injection in the amygdala (*G* and *I*) and food intake after overnight fast and peripheral CNO administration in WT and PKR2^CRE^ male (*H*) and female (*J*) mice transduced with AAV1-hSyn-DIO-hM3(Gq)-mCherry unilaterally into the amygdala (n = 8–9 per group). Results are mean ± SD. ∗*p* < 0.05, ∗∗*p* < 0.01, and ∗∗∗*p* < 0.001. AAV1, adeno-associated virus 1; CNO, clozapine *N*-oxide; gRNA, guide RNA; ICV, intracerebroventricular; PKR, prokineticin receptor.

To determine whether endogenous PK2 plays an important role in controlling food intake, we assessed the effect of central injection of the PKR antagonist PKRA7 on food intake in sated mice. Male and female WT mice cannulated in the lateral ventricle were habituated to BioDAQ cages and ICV injections for 5 days before injection of vehicle or PKRA7 at the beginning of the light phase. Food intake was then recorded for 5 h to determine the effect of PKR2 inhibition on feeding behavior. We observed a significant increase in food intake in both male and female mice injected with PKRA7 compared with control (Fig. 3, *A* and *B*), suggesting that endogenous PK2 plays an important role in controlling energy homeostasis and may prevent overfeeding in sated animals. To further assess the role of PKR2 in PKRA7-mediated increase in food intake, the same experiment was repeated in dCas9-KRAB mice injected bilaterally in the amygdala with AAV1-PKR2 gRNA. In these mice, PKRA7 injection did not increase food intake compared with control (Fig. 3, *C* and *D*), confirming that the orexigenic effect of PKRA7 previously observed (Fig. 3, *A* and *B*) is mediated through PKR2 inhibition.

### Stimulation of PKR2 neurons inhibits food intake

To determine if stimulation of PKR2 neurons is sufficient to inhibit feeding, we specifically expressed the activating DREADD hM3D(Gq) in PKR2-expressing neurons by injecting 5-week-old male and female PKR2^CRE^ mice i.v. with AAV.PHP.eB-hSyn-DIO-hM3D(Gq)-mCherry. PHP.eB AAV viruses can efficiently infect neurons throughout the brain following systemic injection (26). Six weeks after viral injection, overnight fasted mice were injected i.p. with vehicle or with the DREAAD agonist clozapine *N*-oxide (CNO) to stimulate PKR2 neurons. CNO injection resulted in a drastic inhibition of food intake compared with vehicle control in both male and female animals (Fig. 3, *E* and *F*). This result demonstrates that stimulation of PKR2 neurons is sufficient to significantly inhibit food intake.

### Stimulation of LA^PKR2^ neurons is sufficient to inhibit feeding

While the previous experiment demonstrates that activation of PKR2 neurons throughout the central nervous system is potently anorexigenic, it does not identify the brain region responsible for this effect. To test whether stimulation of LA^PKR2^ neurons is sufficient to inhibit food intake, male and female WT (control) and PKR2^CRE^ mice were unilaterally injected in the LA with AAV1-hSyn-DIO-hM3D(Gq)-mCherry to express the activating DREAAD specifically in LA^PKR2^ neurons. Targeting of the virus to the LA was verified by microscopic detection of mCherry in coronal brain sections (Fig. 3, *G* and *I*). Two weeks after virus delivery, animals were fasted overnight and injected with 5 mg/kg CNO i.p. before returning food to cages. Specific unilateral activation of LA^PKR2^ neurons resulted in a significant decrease in food intake both in male and female animals compared with control mice (Fig. 3, *H* and *J*), confirming that stimulation of LA^PKR2^ neurons is sufficient to inhibit food intake.

### MRAP2 regulates PKR2 function *in vivo*

**Figure 4:**
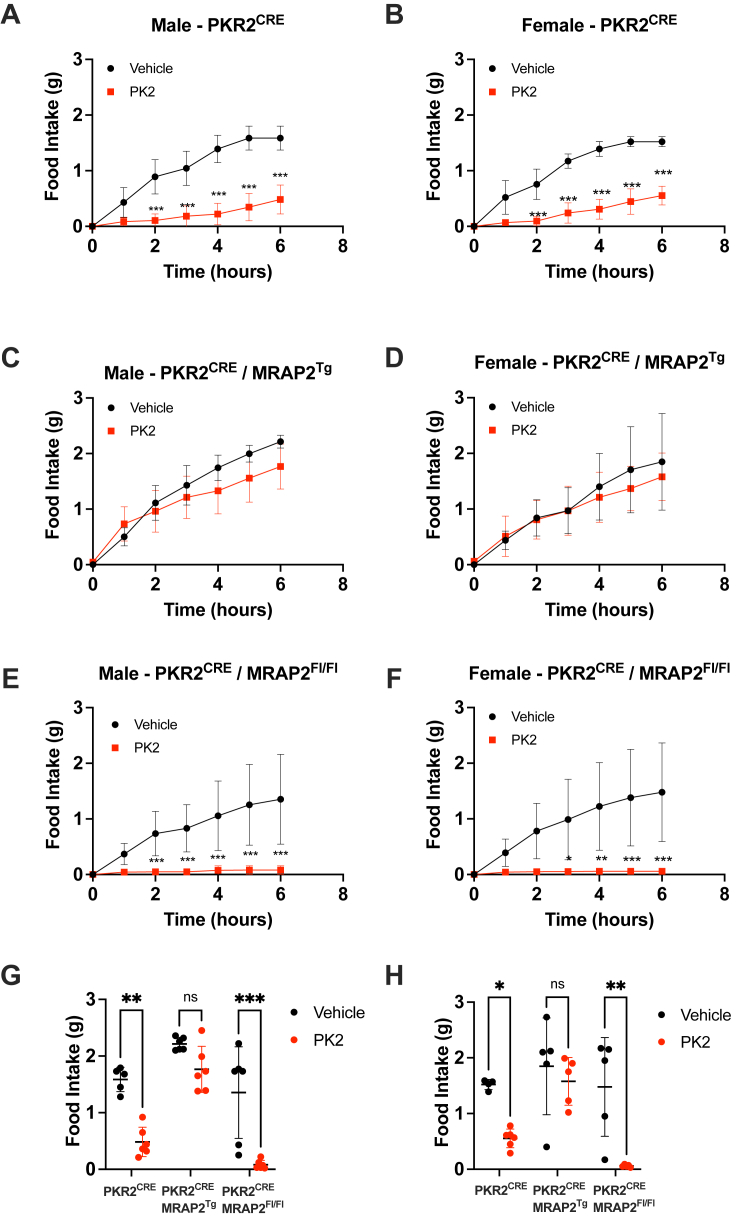
**MRAP2 expression in PKR2 neurons modulates the anorexigenic activity of PK2.***A* and *B*, food intake in overnight fasted male (*A*) and female (*B*) PKR2^CRE^ mice injected ICV with vehicle or 0.65 μg PK2. *C* and *D*, food intake in overnight fasted male (*C*) and female (*D*) PKR2^CRE^/MRAP2^Tg^ mice injected ICV with vehicle or 0.65 μg PK2. *E* and *F*, food intake in overnight fasted male (*E*) and female (*F*) PKR2^CRE^/MRAP2^Fl/Fl^ mice injected ICV with vehicle or 0.65 μg PK2. *G* and *H*, comparison of the cumulative food intake in overnight fasted male (*G*) and female (*H*) PKR2^CRE^, PKR2^CRE^/MRAP2^Tg^, and PKR2^CRE^/MRAP2^Fl/Fl^ 6 h after vehicle or PK2 injection. Results are mean ± SD. ∗*p* < 0.05, ∗∗*p* < 0.01, and ∗∗∗*p* < 0.001. ICV, intracerebroventricular; MRAP2, melanocortin receptor accessory protein 2; PK, prokineticin; PKR, prokineticin receptor.

We have previously shown that MRAP2 regulates the trafficking and signaling of multiple GPCRs involved in the control of energy homeostasis including PKRs 1 and 2 (13, 22). In the case of PKRs, MRAP2 prevents receptor glycosylation resulting in its retention in the endoplasmic reticulum (23, 24). Consequently, MRAP2 decreases the density of PKRs at the plasma membrane and prevents PK2-mediated signaling. To determine if altering MRAP2 expression in PKR2 neurons results in changes in PK2 efficacy, we generated a mouse model that overexpresses a C-terminally V5-tagged MRAP2 in a CRE-dependent manner (MRAP2^Tg^ mouse). This mouse was produced using a vector containing a CAG promoter followed by a floxed 3X-STOP cassette and the coding sequence for MRAP2-V5-IRES-tdTomato (Fig. S2*A*). When bred to PKR2^CRE^ mice, the resulting PKR2^CRE^/MRAP2^Tg^ animals express MRAP2-V5 and tdTomato in PKR2 neurons. Fluorescence imaging of brain slices from PKR2^CRE^/MRAP2^Tg^ mice shows readily detectable tdTomato signal in the LA (Fig. S2*B*), and the overexpressed MRAP2-V5 is detectable by anti-V5 Western blot on lysates from amygdala micropunches (Fig. S2*C*). To determine if deletion or overexpression of MRAP2 in PKR2-expressing neurons results in increased or decreased PK2 efficacy respectively, PKR2^CRE^ mice were bred to MRAP2^Flox^ and MRAP2^Tg^ animals. Male and female PKR2^CRE^/MRAP2^+/+^, PKR2^CRE^/MRAP2^Flox/Flox^, and PKR2^CRE^/MRAP2^Tg^ mice were canulated for ICV injection, allowed to recover, and habituated to BioDAQ home cages. Animals were then fasted overnight and centrally injected with either vehicle or PK2 before providing them with access to food. In male and female PKR2^CRE^/MRAP2^+/+^ mice, PK2 injection caused a very significant inhibition of food intake (Fig. 4, *A*, *B*, *G* and *H*). Overexpression of MRAP2 in PKR2 cells resulted in a dramatic loss of the anorexigenic effect of PK2 in both male (Fig. 4, *C* and *G*) and female (Fig. 4, *D* and *H*) animals, consistent with MRAP2 being an inhibitory accessory protein of PKR2. Furthermore, deletion of MRAP2 in PKR2 neurons resulted in an increased PK2 efficacy with a more pronounced and sustained inhibition of food intake (Fig. 4, *E*–*H*). These results confirm that MRAP2 is an endogenous inhibitory accessory protein of PKR2 and that changes in MRAP2 expression in PKR2 neurons alter their responsiveness to PK2.

### The anorexigenic activity of PKR2 activation is not because of increased anxiety

**Figure 5:**
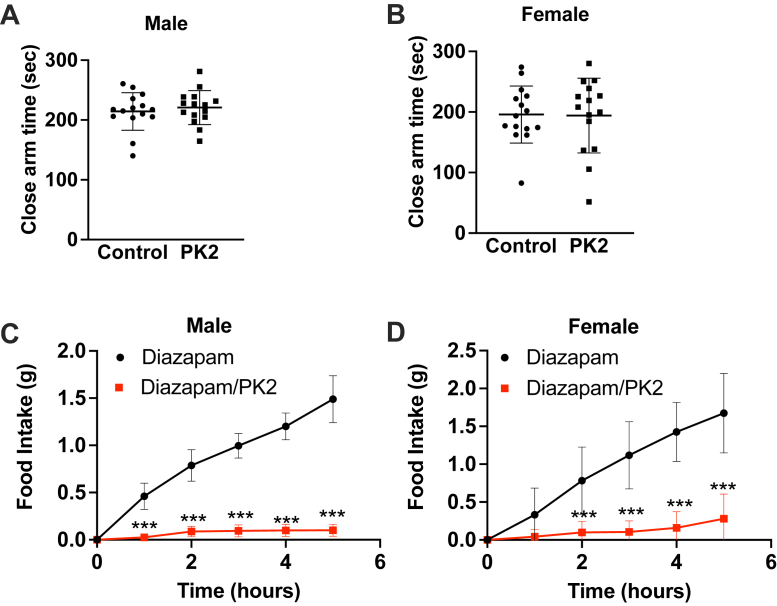
**PK2 is not anxiogenic.***A* and *B*, elevated zero maze test in WT male (*A*) and female (*B*) mice after ICV injection of vehicle or 0.65 μg PK2 (n = 15 per group). *C* and *D*, food intake in overnight fasted WT male (*C*) and female (*D*) mice injected with diazepam i.p. and either vehicle or 0.65 μg PK2 ICV (n = 10–11 per group). Results are mean ± SD. ∗∗∗*p* < 0.001. ICV, intracerebroventricular; PK, prokineticin.

The amygdala is a critical brain region for the regulation of anxiety and stress response (27, 28, 29). For this reason, it is possible that the inhibition of food intake following activation of LA^PKR2^ neurons is a response to an anxiety stimulus rather than a satiety signal. To determine if central activation of PKR2 results in increased anxiety, male and female mice were canulated in the lateral ventricle and allowed to recover from the surgery. Animals were then injected ICV with either vehicle or PK2 and placed on an elevated zero maze. During the 10 min test, animals were tracked, and time spent in open and closed areas was quantified. PK2 did not increase the time spent in the closed area in either male (Fig. 5*A*) or female (Fig. 5*B*) mice, thus suggesting that activation of PKRs is not anxiogenic. To further test whether increased anxiety contributes to the anorexigenic effect of PK2, we measured food intake after an overnight fast in male and female mice centrally injected with vehicle or PK2 after an i.p. injection of the anxiolytic diazepam. Diazepam treatment did not impair the anorexigenic effect of PK2 (Fig. 5, *C* and *D*), thus further demonstrating that the anorexigenic effect of the PKR2 agonist is not caused by increased anxiety.

## Discussion

PKRs have been shown to regulate multiple physiological functions, including nociception, reproductive function, and energy homeostasis. PKs were first identified as factors enhancing gastrointestinal smooth muscle contractility but were rapidly shown to cause hyperalgesia and potently decrease food intake. Two GPCRs, PKR1 and PKR2, can be activated by PKs and PKR1, largely because of its expression in the ARC, was originally hypothesized to be responsible for the regulation of appetite. However, the contribution of PKR1 and PKR2 on the anorexigenic effect of central PK2 injection had not been tested. For this reason, we generated a *Pkr1* KO mouse model and found that deletion of PKR1 does not impair the ability of central PK2 to decrease food intake, thus suggesting that PKR2 is responsible for PK2-mediated regulation of energy homeostasis. Whereas *Pkr2* KO mice have previously been generated, their phenotype mimicking the Kallmann syndrome make them a poor model to study the role of PKR2 in the regulation of food intake. Global *Pkr2* KO animals display hypoplasia of the olfactory bulb and atrophy of the reproductive system. In this study, we demonstrate that silencing of PKR2 expression in the amygdala of adult animals using CRISPR interference technology results in a clear impairment of the anorexigenic function of PK2, thus consistent with PKR2 being responsible for mediating the effect of PK2 on energy homeostasis. In addition, our results suggest that the inhibition of food intake by PK2 is not mediated through neurons of the ARC but rather through neurons within the LA. This is supported by the fact that whereas the ARC-targeted silencing of PKR2 does not result in a loss of PK2 activity, silencing of PKR2 expression in the LA is sufficient to abrogate PK2-mediated feeding inhibition. We also show that global or LA-targeted DREADD-mediated activation of PKR2 neurons results in feeding inhibition. Expressing activating DREAD in all PKR2 neurons resulted in a larger inhibitory effect on feeding than LA^PKR2^ targeted DREADD, which could mean that PKR2 neurons in other areas of the brain also contribute to the regulation of food intake. It is however possible that the smaller effect observed in mice with LA^PKR2^ neurons targeted DREAAD was due to the unilateral virus delivery, thus only engaging half of the LA^PKR2^ neurons. The amygdala, which is a major cite for the control of fear and anxiety, is emerging as an important regulator of energy homeostasis with the ability to respond to multiple satiety and anorexigenic cues. The anorexigenic response to PKR2 stimulation in the amygdala is not because of increased anxiety and thus represent a *bona fide* pathway that controls energy homeostasis. The finding that PK2 acts through activation of neurons located in the amygdala independently of hypothalamic circuits may explain our previous finding that PK2 can decrease food intake in *Mc4r* KO mice. Identifying neuronal networks projecting to and from amygdala neurons involved in feeding regulation, including PKR2 neurons, is important and may uncover novel approaches to modulate hunger and to treat obesity.

## Experimental procedures

### Animals

C57BL/6NJ (Jax strain: 005304) and dCas9-KRAB (Jax strain: 030000) were purchased from Jackson Laboratory. *Pkr1* KO and *Mrap2*^*Tg*^ mice were generated by the genome editing core facility at the University of Iowa. *Pkr2*^CRE^ mice were a generous gift from Dr Carol Elias (University of Michigan) (10). *Mrap2*^flox^ mice were generated by the Sanger Mouse Genetics Project. All mice used are on a C57BL/6NJ background. All animals were maintained at the University of Iowa temperature-controlled animal facility with 12 h light/dark cycles (6 AM/6 PM). Animals were fed with standard rodent diet (NIH-31) and allowed free access to water. All experiments using mice were approved by the Animal Care and Use Committee at the University of Iowa.

### Viruses

AAV1-GFP was purchased from the University of Iowa Viral Vector Core. pAAV1-hSyn-DIO-hM3D-mCherry and pAAV.PHPeB-hSyn-DIO-hM3D-mCherry were purchased from Addgene (#44361), and AAV1-PKR2 gRNA was produced by cloning a gblock containing the sequence for PKR2 gRNA1-U6 promoter–PKR2 gRNA2 (GCGACACACGCCCACCAAGTGTTTTAGAGCTAGAAATAGCAAGTTAAAATAAGGCTAGTCCGTTATCAACTTGAAAAAGTGGCACCGAGTCGGTGCTTTTTTGGCCGGCCGAGGGCCTATTTCCCATGATTCCTTCATATTTGCATATACGATACAAGGCTGTTAGAGAGATAATTGGAATTAATTTGACTGTAAACACAAAGATATTAGTACAAAATACGTGACGTAGAAAGTAATAATTTCTTGGGTAGTTTGCAGTTTTAAAATTATGTTTTAAAATGGACTATCATATGCTTACCGTAACTTGAAAGTATTTCGATTTCTTGGCTTTATATATCTTGTGGAAAGGACGAAACACCGAAGACTGATGCGAGGAGAGC) in to the SapI-digested AAV-U6-sgRNA-hSyn-mCherry plasmid (Addgene; #87916) using HiFi DNA Assembly Kit (NEB). AAV1 particules were produced in human embryonic kidney 293 cells and purified using the AAVpro Purification Kit Maxi (Takara; catalog no.: 6666).

### Cannula implantation

Animals were anesthetized with 2.5% isoflurane inhalation before being placed on a stereotaxic apparatus (David Kopf Instruments). After standard disinfection of surgical site, an incision was made to expose the skull, and a small hole was drilled. A 22-gauge stainless steel guide cannula was implanted to targeted brain regions. For ICV injections, guide was placed anterior/posterior (A/P)—0.5 mm, medial/lateral (M/L)—1.0 mm, dorsal/ventral (D/V)—1.5 mm from brain surface. For amygdala injection, guide was placed A/P—1.37 mm, M/L—3.37 mm, and D/V—3.67 mm from brain surface. Guide cannula was fixed in place with dental cement, and mice were allowed to recover for 2 weeks postsurgery before experiment.

### Virus injections

Mice were anesthetized by 2.5% isoflurane inhalation, and injection of 1 μl of virus was targeted to the ArcN of the hypothalamus (A/P—1.6 mm, M/L—0.2 mm, and D/V—5.4 mm) or to the amygdala (A/P—1.37 mm, M/L—3.37 mm, and D/V—3.67 mm) with a flow rate of 200 nl/min. At completion of virus injection, needle was kept in injection for an additional 15 min. For PHPeB viruses, 4-week-old mice were injected iv with 1 × 10^9^ viral particles retro-orbitally. Mice recovered from surgery for at least 2 weeks before experimentation.

### DREADD experiments

Mice expressing DREADDs were injected with vehicle or 5 mg/kg of CNO i.p.

### BioDAQ feeding studies

About 10- to 12-week-old mice were used for feeding studies using the BioDAQ food intake monitoring system (Research Diets). Mice were placed in the BioDAQ home cages for 5 to 7 days to allow for acclimation to the new environment and daily handling. For fast refeed experiments, food access was blocked overnight before performing experiments. Food intake was continuously measured by the BioDAQ system.

### qPCR

Mice were euthanized, and punches of amygdala or ARC were collected and snap frozen in liquid nitrogen. RNA was isolated with TRIzol reagent according to the manufacturer’s protocol (Invitrogen; catalog no.: 15596026). About 1 μg of RNA was used to generate complementary DNA according to the manufacturer’s protocol (Applied Biosystems; catalog no.: 4387406). PKR2 mRNA expression was quantified using Taqman qPCR with PKR2 and βActin primer/probe sets (PKR2—IDT 260920495; βActin—IDT 210598000). Reactions were performed in a QuantStudio 3 Real-Time PCR System (Applied Biosystems).

### Fluorescence imaging

Animals were deeply anesthetized with isoflurane and perfused with ice-cold PBS followed by ice-cold 4% paraformaldehyde in PBS. Whole brains were dissected and post fixed in fresh 4% paraformaldehyde for 12 h at 4 °C. Brains were immersed in 30% sucrose in PBS at 4  °C until sunk. Brain was coroMnally sectioned (40 μm) using a sledge microtome (Leica Biosystems). Free-floating sections were mounted onto SuperFrost slides (Fisher Scientific), air-dried at room temperature, and coverslipped with Vectashield hardset antifade mounting media with 4′,6-diamidino-2-phenylindole (catalog no.: H-1500). Slices were imaged using the Olympus IX3 microscope system and stitched together with the Olympus Cellsens Dimension software.

### Zero maze

Twelve-week-old mice cannulated in the lateral ventricle were centrally injected with vehicle or 0.65 μg PK2 prior to being placed on Zero Maze platform (Stoelting). Mice freely explored the maze for 10 min, and the time spent in the open/close area was quantified using ANY-maze software (Stoelting).

### Statistical analysis

Statistical analyses were conducted with GraphPad Prism 9.3.1 (GraphPad Software). The data are shown as mean ± SD. Two-way repeated-measures ANOVA followed by post hoc Sidak’s multiple comparisons test or unpaired *t* test was used to determine statistically significant differences between the groups tested time points and different samples.

## Data availability

All the data are contained in the article.

## Supporting information

This article contains supporting information.

## Conflict of interest

The authors declare that they have no conflicts of interest with the contents of this article.
