# Supplementary figures and images for: Activation of amygdala prokineticin receptor 2 neurons drives the anorexigenic activity of the neuropeptide PK2
Source: J Biol Chem. 2022 Dec 17;299(1):102814. doi: 10.1016/j.jbc.2022.102814 (PMC9860486; doi:10.1016/j.jbc.2022.102814)

**A**

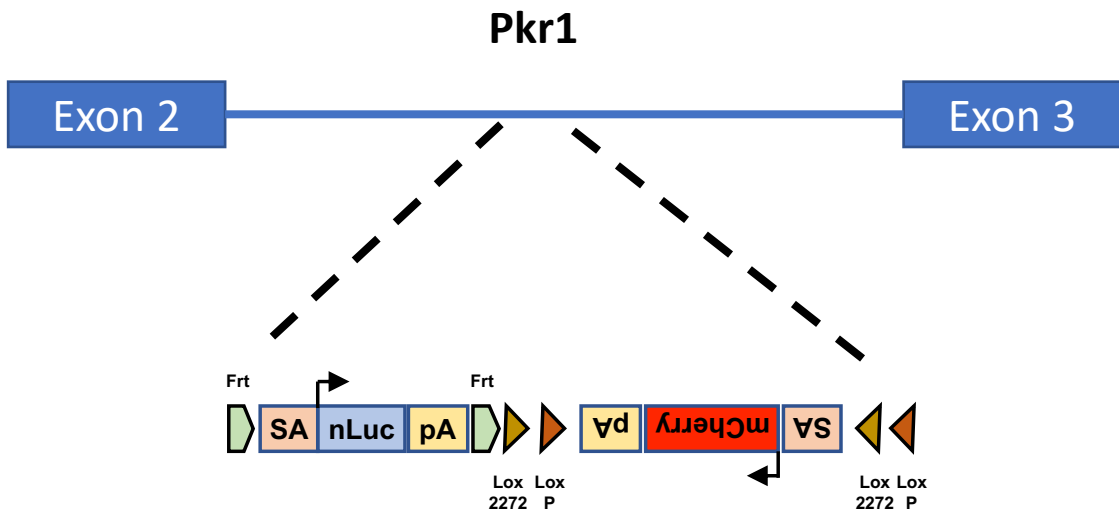

**B**

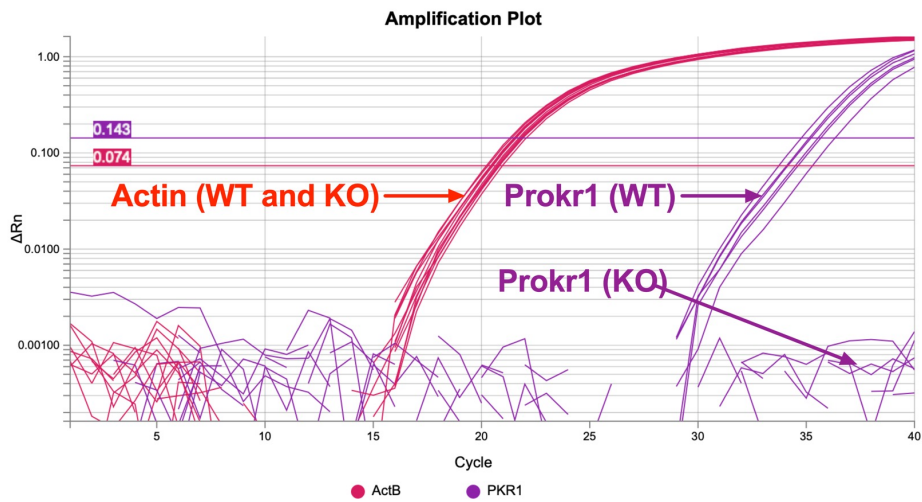

**C**

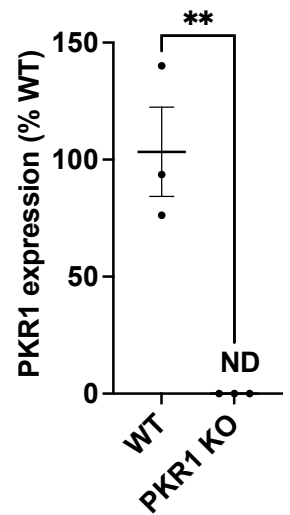

Supplement: Figure S1 [file mmc1.pdf]

**A**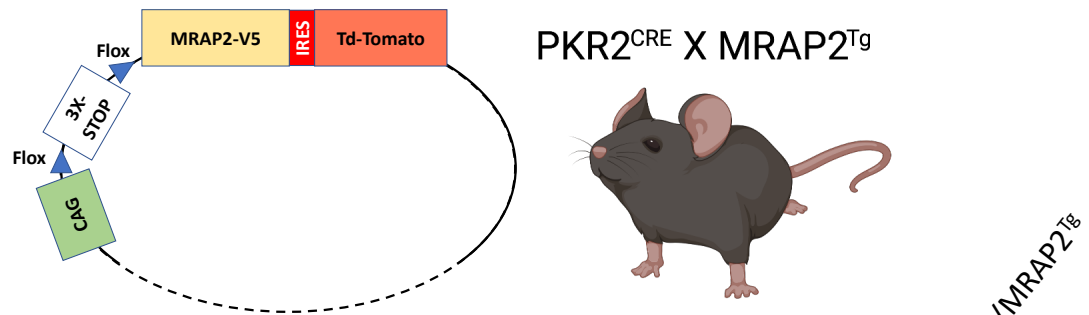**B**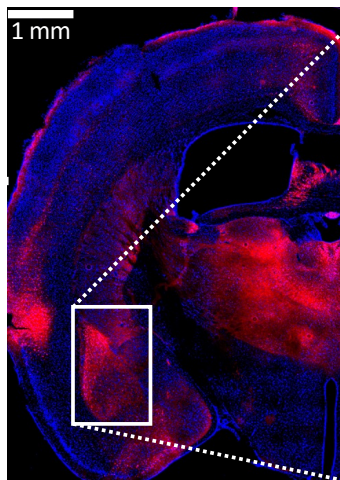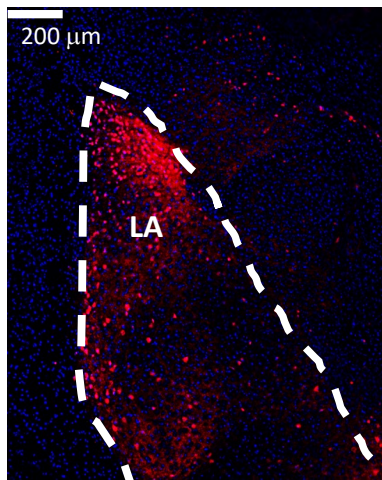**C**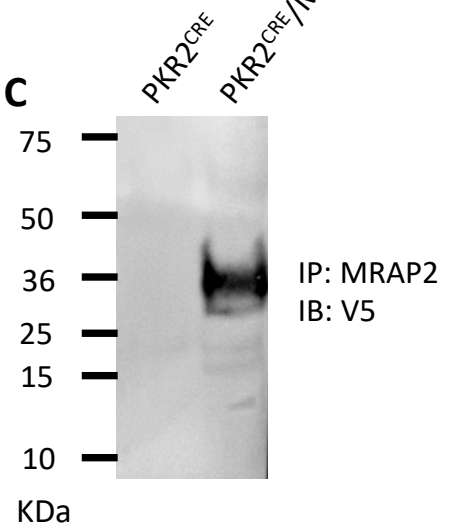

Supplement: Figure S2 [file mmc2.pdf]
